# Participation in Everyday Activities of Children with and without Neurodevelopmental Disorders: A Cross-Sectional Study in Spain

**DOI:** 10.3390/children7100157

**Published:** 2020-10-01

**Authors:** Nerea Blanco-Martínez, Laura Delgado-Lobete, Rebeca Montes-Montes, Nuria Ruiz-Pérez, Marcos Ruiz-Pérez, Sergio Santos-del-Riego

**Affiliations:** 1 Faculty of Health Sciences, Health Integration and Promotion Research Unit (INTEGRA SAÚDE), University of A Coruña, 15011 A Coruña, Spain; nerea.blanco1@udc.es (N.B.-M.); sergio.santos.delriego@udc.es (S.S.-d.-R.); 2 Faculty of Educational Sciences & Sports, University of Vigo, 36005 Pontevedra, Spain; 3 TALIONIS Research Group, Centre for Information and Communications Technology Research (CITIC), University of A Coruña, 15008 A Coruña, Spain; rebeca.montes@udc.es; 4 Faculty of Pharmacy, University of Santiago de Compostela, 15782 Santiago de Compostela, Spain; nuriamaria.ruiz@rai.usc.es; 5 Faculty of Health Sciences, Catholic University of Ávila, 05005 Ávila, Spain; 6 University College of Teacher Training, University of Vigo, 36214 Vigo, Spain; marcos91rp@gmail.com

**Keywords:** neurodevelopmental disorders, autism spectrum disorders, attention deficit and hyperactivity disorder, motor coordination disorder, activities of daily living, participation, occupational therapy

## Abstract

Children with neurodevelopmental disorders (NDDs) often report significant difficulties performing activities of daily living (ADLs), which may restrict their daily participation. The aim of this study was to investigate the differences in ADLs participation between children with NDDs and typically developing (TD) children, and to explore the associations between different daily participation contexts. A cross-sectional study was conducted that included twenty children with a medical diagnosis of an NDD and 26 sex- and age-matched TD controls. The daily participation across home, community, school, and instrumental living activities was measured using the Child and Adolescent Scale of Participation (CASP). The results show that children with NDDs engaged in lower participation in all CASP contexts (Δ = 1.7–5.5, *p* < 0.001) and had a significantly higher prevalence of moderate or severe restricted participation than their TD peers (OR = 23.4, 95% CI = 3.6–154.2, *p* < 0.001). Additionally, a strong association was found between the different contexts of participation (*r* = 0.642–0.856). Overall, the children with NDDs experienced significant participation restrictions on their daily activities. This study adds to the growing evidence showing that intervention strategies in this population should adopt a participation-oriented approach.

## 1. Introduction

Neurodevelopmental disorders (NDDs) are a group of conditions with onset in the early developmental period and comprise a broad group of developmental deficits in the brain function that affect physical, social, academic, and occupational functioning [1,2]. These disorders constitute the most frequent conditions for disability and participation restriction during childhood and are more frequent in males than females [3]. The estimated prevalence of NDDs in school-aged children worldwide ranges from 4 to 13%, but these data vary across countries and estimation methods [3,4,5,6]. In most of the cases, the NDDs persist into late adolescence and adulthood, with persistent consequences for daily living functioning [6]. The most prevalent and usually reported NDDs in childhood are attention deficit and hyperactivity disorder (ADHD), developmental coordination disorder (DCD), and autism spectrum disorders (ASDs). In a comprehensive review on ADHD prevalence conducted by Polanczyk et al., it was reported that 9.5% of children had a lifetime diagnosis of ADHD worldwide during the past three decades [7], while this prevalence ranges between 4.1% and 8.8% among Spanish children and adolescents [8,9,10]. Reported estimates of the prevalence of DCD in schoolchildren usually range between 5 and 6% [11,12,13] but recent studies have found that up to 12.0% of Spanish children are at risk of DCD [14,15]. While the prevalence estimates of ASDs are significantly lower, with approximately 1–2% of children presenting with an ASD, the consequences of these disorders in everyday living are much more severe and restrictive, thus making ASDs some of the most studied neurodevelopmental conditions [9,16,17,18,19]. Overall, approximately 9.4% of Spanish children have a neurodevelopmental condition that interferes with their functional development [9].

Children with NDDs face significant difficulties in activity performance from early childhood onward, particularly in self-care activities, play and mobility, social cognition, and instrumental activities [20,21,22,23,24]. However, not only activity performance is restricted in these children but daily participation is compromised as well. Participation in meaningful activities of daily living (ADLs) is defined as involvement in life contexts or situations, where the International Classification of Functioning, Disability and Health (ICF) of the World Health Organization consider this aspect to be a crucial part of children’s healthy psychological, emotional, and functional development [25].

Additionally, satisfactory participation in a particular daily context is hypothesized to be intimately related to participation in other daily contexts, and thus, restrictions or difficulties in one occupational area are expected to lead to restrictions in other activities of daily living as well [26,27,28]. This hypothesis is supported by research that shows an interrelation in participation between different daily contexts in typically developing (TD) children [29,30,31]. Moreover, performance difficulties or participation restrictions are rarely present in only one particular area but are reported in most daily contexts and activities [32].

Several studies have reported that motor, social, behavioral, and processing impairments of children with NDD lead to participation restrictions, and that this population is more likely to show lower and less meaningful participation in daily contexts than typically developing children [32,33,34,35,36]. Three literature reviews using the ICF have concluded that more studies should be conducted to explore the consequences of ADHD, DCD, and ASDs in the context of participation restrictions [35,36,37]. Investigating potential limitations in daily, naturalistic contexts is relevant for intervention strategies as well, as scientific research shows that activity- and participation-oriented approaches should be used to promote daily performance and family participation in children with neurodevelopmental conditions [11,38,39,40,41,42]. However, very few studies have focused on daily participation differences in Spanish children with and without NDDs. To the best of our knowledge, only two studies have explored social participation among children with ADHD, ASDs, or co-occurring disorders in Spain [43,44], but participation in self-care, instrumental, or community contexts was not assessed. Thus, little is known about daily participation restrictions in Spanish children with neurodevelopmental conditions. Additionally, exploring how children with and without typical development perform and participate during their daily activities is crucial for designing meaningful interventions [45].

Daily participation is influenced by cultural and country constraints, where this effect may differ between participation contexts, even between similar European countries. For instance, a recent study found significant differences in daily participation in self-care activities between typically developing Spanish and Dutch children [12]. Another study found that Spanish boys participated less than German boys in self- and house-care activities [46]. While this research focused on typically developing children, it was expected that differences in daily participation patterns would be found in children with neurodevelopmental conditions as well. As there is no information regarding differences in daily participation in Spanish children with and without NDDs, a study that evaluated participation restrictions between both groups in Spain was needed. Therefore, the aims of this study were (a) to explore differences in participation between Spanish children with and without NDDs and (b) to explore the association between participation in different daily contexts.

## 2. Materials and Methods

### 2.1. Research Ethics, Procedure, and Participants

A multicenter, comparative cross-sectional study was carried out. This study was approved by the Autonomic Research Ethics Committee of Galicia, Spain (code 2018-544). All participants consented to take part in the study anonymously and confidentially.

The total sample comprised forty children aged 5 to 12 years. This sample size was estimated in order to measure the differences in ADLs participation between children with and without developmental disorders with a minimal bias (α < 0.01, power (1 − β) > 0.99) [31]. Children with NDDs (*n* = 20) were recruited from two private rehabilitation centers in Vigo and A Guarda (Spain). Children in this group had a medical diagnosis of a neurodevelopmental condition (girls = 35.0%; mean age = 7.8 years, standard deviation (SD) = 1.7 years; ASDs = 50.0%, motor coordination/psychomotor disorders = 25.0%, ADHD = 15.0%, pervasive developmental disorder/not otherwise specified = 10.0%). A second sample of children without developmental or learning disorders (TD group; *n* = 20) with a similar sex and age distribution was recruited to serve as a sex- and age-matched control group (girls = 35.0%; mean age = 8.7 years, SD = 1.9 years). Children in this group attended three different mainstream schools in Vigo (Spain) and were excluded beforehand if their parents reported a medically diagnosed neurodevelopmental condition. Table 1 shows the sociodemographic characteristics of both groups. Children of both groups were similar in age, sex distribution, education track, and type of school.

Parents of the participants received the Child and Adolescent Scale of Participation (CASP) questionnaire as well as an informative letter about the study between December 2018 and June 2019 via school or rehabilitation center intermediation. The letter included the e-mail address and telephone number of the first author such that parents could reach the research team for clarification of the study or the questionnaire. Only parents who consented to anonymously participate completed and returned the CASP to the schools or rehabilitation centers from where they were retrieved by the first author.

### 2.2. The Child and Adolescent Scale of Participation

The Spanish parent-reported version of the CASP was used to measure daily participation and participation restrictions [31,47]. This scale is part of the Child and Family Follow-up Survey, which is a parent-reported measure informed by the ICF that was originally developed by the occupational therapist Gary Bedell to monitor the needs and outcomes of children and adolescents with an acquired brain injury and their families. The CASP has been validated in multiple settings across different countries and contexts with children and adolescents with other conditions, including NDDs [31]. The CASP has reported an excellent internal consistency and temporal stability (Cronbach’s alpha = 0.96; intraclass correlation (ICC) = 0.94) and a good convergent validity with the Pediatric Evaluation of Disability Index (*r* = 0.51–0.75) and the Child and Adolescent Scale of Environment (*r* = 0.43–0.57) [31].

The CASP consists of 20 items and it is available in both parent-reported and self-administered versions. The purpose of this scale is to measure the extent of participation and participation limitations of children and adolescents in comparison to an age-expected performance in four different contexts (home = six items, community = four items, school = five items, and home and community living activities = five items) [31,47].

While “home” and “community” scales cover social-oriented or self-maintenance activities within the home and community, respectively, the “home and community living activities” scale refers to those activities that support daily life within the home and community, which often require more complex tasks and interactions (i.e., instrumental activities) [26,31]. In the present study, we did not include item 20—work activities and responsibilities (e.g., completion of work tasks, punctuality, attendance, and getting along with supervisors and co-workers)—as the Spanish legal working age is 18 years, and therefore none of the children enrolled in the study were expected to participate in that activity. This decision was made based on the recommendation of two Spanish occupational therapists who independently revised the items.

Each item describes a daily activity and it is rated on a four-point Likert scale (age expected = 4, somewhat limited = 3, very limited = 2, unable to participate = 1). An additional response of “not applicable” is available if the item is not appropriate for the child’s age, and items rated as “not applicable” do not receive a score. The item scores are summed and divided by the maximum possible score based on the number of items scored. This score is then multiplied by 100 such that total scores for the subscales and total scale range from 0 to 100, where higher scores indicate a greater level of participation [48].

In addition to the standard analysis of the CASP, two different item-level score analyses of the scale were conducted in the present study to analyze the severity of participation restrictions in each context and for global ADLs [48]. First, item scores of 1, 2, or 3 were considered to explore mild or moderate participation limitations between groups. For instance, if a child was rated as “participation somewhat limited” or lower in at least one activity, they would be defined as presenting with at least mild participation restrictions. Second, we explored moderate or severe participation limitations by considering item scores of 1 or 2. In these cases, if a child was rated as “participation very limited” or “unable to participate,” they would be defined as presenting with moderate or severe participation restrictions. Therefore, using the standard analysis of the CASP we could compare the level of daily participation between children with and without NDDs, while using the categorized item-level scores, we could explore how many children showed mild-to-severe participation restrictions in each context.

### 2.3. Data Analysis

The sample size estimation was performed using G*Power version 3.1.9.4. (Heinrich-Heine-Universität Düsseldorf, Düsseldorf, Germany) [49]. The statistical analyses were conducted using SPSS version 25 (SPSS Inc., Chicago, IL, USA). Prior to conducting the analysis of the data, the internal consistency of the CASP in the sample was tested using Cronbach’s alpha to ensure that the removal of item 20 did not alter the reliability of the questionnaire. Values of 0.7 or higher were considered indicators of good internal consistency. The data were examined to determine whether it had a normal distribution using visual inspection, skewness, and kurtosis [50].

Student *t*-tests for independent samples were used to analyze the differences in participation scores in the CASP total and subscale scores between children with and without NDDs. The effect size of these differences was estimated with Glass’s delta (Δ) using the standard deviation of the typically developing group [51]. Differences in the prevalence of participation restrictions in the different contexts and global participation between both groups were analyzed using chi-squared tests. Additionally, the odds ratios (ORs) and OR 95% confidence of intervals (95% CIs) were calculated to estimate the risk for participation restrictions between children with and without NDDs [51]. Finally, the association between the different contexts of participation was examined in both the total sample and within groups using Spearman correlation coefficients.

## 3. Results

The internal consistency values of the CASP were adequate for both the overall sample (Cronbach’s alpha = 0.9) and for the two groups of participants (TD = 0.7, NDDs = 0.9). Most of the items were scored, indicating that the activities were relevant for the child, and only item 9, item 14, and item 19 received no applicable scores (*n* = 1, *n* = 1, and *n* = 14, respectively). There were no significant differences in the no applicable scores in those items between both groups (*p* = 0.311–0.320).

As shown in Table 2, significant and strong differences were found in all contexts of participation between children with and without NDDs. Community participation, home participation, and general participation were the contexts that revealed stronger differences between groups (Δ = 4.0–5.5, *p* < 0.001).

Children with NDDs showed a higher prevalence of participation restrictions or limitations in all contexts (*p* < 0.01). While up to 35.0–60.0% of children with typical development reported a mild or moderate participation limitation in at least one ADL (see Table 3), all the children in the NDDs group reported moderate-to-severe limitations or were unable to participate in at least one instrumental ADL, and most of them faced participation restrictions in home, community, and school settings as well (Table 3). Overall, children with NDDs were 23.4 times more likely to suffer significant participation limitations during their daily living.

Finally, the Spearman correlations exposed significant and moderate-to-strong associations between the different contexts of participation in the total sample and within the NDDs group (Table 4). The TD group showed significant correlations between community and school participation, and between community and home and community instrumental living activities participation (*r* = 0.484–0.604), although the correlation between home and community participation was close to significance (*p* = 0.068). The strongest correlations were found between school and home participation, and between school and community participation in both the total sample and the NDDs group (*r* = 0.641–0.856).

## 4. Discussion

Neurodevelopmental conditions that affect a child’s typical development are present in approximately 9% of school-aged children in Spain [9]. These children usually face more performance difficulties and participation restrictions than their TD peers, which might have further consequences on their emotional and psychological wellbeing [20,21,22,23,24,32,33,34,35,36,37,38]. To the best of our knowledge, no studies on NDDs have so far compared participation restrictions in the different daily contexts in Spanish children with and without NDDs.

### 4.1. Differences in Daily Participation between Children with and without NDDs

Significant differences in daily participation between children with and without neurodevelopmental conditions were found in the present sample. As expected, children with ASDs, motor coordination disorders, or ADHD participated significantly less and faced more participation restrictions in all daily contexts than TD children. Such a pattern is consistent with previous research [32,33,34,35,36,37,38]. Our results regarding the level of participation in daily contexts between children with and without developmental disorders are highly similar to the findings of Bedell in the validation study for the CASP [31], who found significant differences between both groups with an analogous outcome in children from North America, Australia, and Israel. Additionally, children with ADHD, DCD, or ASDs usually have co-occurring sensory processing issues [44,52,53], which may further restrict their daily participation [12,44,53,54,55,56]. However, it is worth noting that children with neurodevelopmental conditions who are not currently referred to rehabilitation intervention may have different participation patterns. Although most of the NDD children in this study were enrolled in ordinary education, other children with NDDs who are not in inpatient treatment could also show potential differences in their participation and performance [52].

In the present study, participation restrictions in self-care and instrumental ADLs in home and community contexts were significantly higher in children with NDDs. These activities are oriented toward taking care of one’s own body and supporting daily life within the home and community [26]. Our findings in these contexts are in line with previous studies that explored children from Europe, America, and Asia, which is of particular interest given that self-care and home maintenance activities may be different between cultures. Self-care and instrumental activities are one of the most problematic contexts for children with ASDs, ADHD, and DCD [21,24,33,35]. In addition, van der Linde et al. found that children with DCD both participated significantly less and performed significantly worse than TD children in self-care activities, while daily performance and participation were interrelated as performance predicted participation [23].

In our sample, children in the NDDs group showed lower levels of participation in the school context. Previous studies have highlighted the specific school-related issues that children with developmental disabilities encounter during their daily performance. Difficulties in numerical and mathematical comprehension, reading, academic fine motor activities, and peer socialization have been reported in children with motor coordination problems [33,57,58]. Children with ASDs face significant issues with learning processing and social participation, which negatively affect their academic performance and participation [44,59]. In addition, children with ADHD are more likely to face academic difficulties and to not pursue high school or university studies due to behavioral and attention deficits [44,60].

Social participation in children with neurodevelopmental disabilities is one of the most restricted contexts and one major concern for the families of these children. While the CASP does not consider a specific context of social participation, this aspect is thoroughly evaluated in all subscales, especially in the home and community participation contexts [31,47], which were significantly restricted in the NDDs group. This outcome is consistent with previous research. While exploring the level of participation in several leisure and social activities, Kaljača et al. found that children with neurodevelopmental disabilities not only participated significantly less than TD children, but also that their activities were mainly stereotypical and highly structured, and were mostly supervised by parents [61]. Social cognition has been reported to be limited in these children as well, and it is associated with motor parameters, which further restricts daily participation [20,24,62]. Similarly, in the study conducted by Kreider et al., the social networks and participation with others of children with ASDs, ADHD, and learning disorders were explored [63]. The study concluded that children with neurodevelopmental conditions show specific limitations in physical, recreational, social, and informal activities with family and/or friends.

Children’s participation in family activities in home and community contexts was significantly more restricted in children with NDDs. This finding adds to the evidence that family involvement and functioning is affected in families with children with NDDs, which was previously reported [56,64,65,66]. Family engagement in the child’s activities is important for their overall development, quality of life, and participation involvement [67,68]. Overall, family support is essential for promoting ADLs performance and participation, as parental self-efficacy and satisfaction predict children’s participation involvement and enjoyment [67,69].

An interesting finding of the present study was that 35% of the overall parents (TD = 25%, NDDs = 45%, *p* = 0.320) considered the last item of this context (item 19—using transportation to get around in the community, e.g., to and from school, work, social, or leisure activities) as not applicable, meaning that their child was not expected to participate in that particular activity regardless of the presence of a neurodevelopmental condition. While this could be related to the young age of the participants (<12 years), it would be interesting to examine the subjective relevance perceived by the children about engaging in different daily contexts.

### 4.2. Associations between the Daily Contexts of Participation

Finally, all daily contexts were strongly associated with each other in our sample. This finding suggests that daily participation is a complex and multifaceted construct comprised of several subareas of different but interrelated occupations, as it has been proposed by previous research [26,30]. Interestingly, school participation showed the strongest correlations with both home and community participation in the total sample and the NDDs group while being the context where children with NDDs faced greater participation restrictions. Additionally, correlations between contexts were different between children with and without NDDs, with them being significantly higher in the latter group. This particular result may suggest that participation restrictions in one daily context may have a greater influence on participation restrictions in other contexts in children with neurodevelopmental conditions in comparison with their typically developing peers, and therefore it may be necessary to pay particular attention when these children first show participation restrictions in their daily activities. Overall, these findings add to the existing literature recommending the use of family-centered and occupation- and participation-based interventions, such as occupational therapy in schools [70,71,72,73]. This is of particular relevance given than most children with DCD or ADHD are enrolled in ordinary schools, which do not usually provide this kind of intervention in Spain [52].

Overall, the findings from this study support that children with NDDs present with lower levels of daily participation and greater and more severe participation restrictions in their daily activities than typically developing children. This outcome is consistent with previous research from different countries and cultures, and altogether may point toward a cross-cultural model of participation restrictions in children with NDDs. Additionally, our results suggest that participation difficulties in one particular context may lead to participation difficulties in other contexts, especially in children with neurodevelopmental conditions. Therefore, these findings have implications for research on neurodevelopmental disabilities, both in Spain and in international populations, and they could be used on clinical intervention to assess for difficulties in overall daily participation as soon as a child shows participation restrictions in one particular area, such as school or self-care contexts.

### 4.3. Limitations and Future Research Directions

This study has several limitations that need to be disclosed. First, a limited sample size was used; although this sample size was calculated to examine differences in daily participation between children with and without NDDs with minimal bias [31], the study findings should be interpreted cautiously and should be supported by larger studies. Nevertheless, this. Second, it is important to note that most of the children in the NDDs group had ASDs, which is usually associated with greater daily challenges than other conditions like ADHD or DCD. This could partially explain the greater variance of the participation abilities observed in this group. Additionally, this imbalance in diagnosis distribution within the NDDs group does not allow for a more in-depth analysis of potential differences between the different diagnostic groups. Third, the participants had a large age range (5 to 12 years). Even though both groups had a similar age distribution, differences in daily participation according to age could not be explored due to the limited sample size; therefore, this may pose a further limitation that should be explored in future studies. Additionally, there are other factors that could further restrict daily participation, such as psychosocial and behavioral issues, which are present in a large percentage of children with NDDs [34,35,36,37,74,75]. Finally, we used a parent-reported questionnaire to assess daily participation, which could be subject to subjectivity. However, parental questionnaires can provide valuable information that would not have been recorded otherwise in a clinical setting [76,77], and therefore parent-based measures, such as the CASP, are useful for reporting information about children’s participation in the daily, naturalistic contexts. Future studies should assess differences in participation between larger samples of children with different neurodevelopmental diagnoses that involve considering other environmental, psychosocial, and child-related factors that may be influencing daily participation.

## 5. Conclusions

Findings from this study support the belief that participation difficulties and restrictions are present in Spanish children with NDDs in comparison with their TD peers. Home, community, and school contexts seem to be particularly affected, which may further restrict the quality of life and future development of children with neurodevelopmental issues. These consequences for daily functioning highlight the need for tailored, participation-oriented intervention strategies.

## Figures and Tables

**Table 1:** Sociodemographic characteristics of the sample (*n* = 40).

Sociodemographic Variables	TD Group (*n* = 20)	NDDs Group (*n* = 20)	*p*-Value
Age (mean (SD))	8.7 (1.9)	7.8 (1.7)	0.107
Sex (*n* (%))			1.000
Boys	13 (65.0)	13 (65.0)	
Girls	7 (35.0)	7 (35.0)	
Type of education track (*n* (%))			0.311
Ordinary	20 (100.0)	19 (95.0)	
Ordinary–special combined	0 (0.0)	1 (5.0)	
Type of school (*n* (%))			0.197
Public	10 (50.0)	14 (70.0)	
Semiprivate	10 (50.0)	6 (30.0)	
Age at diagnosis (in months, mean (SD))	NA	37.7 (17.1)	NA
Professional that conducted the NDD diagnosis (*n* (%))	NA		NA
Neurologist		13 (32.5)	
Psychiatrist or clinical psychologist		5 (12.5)	
Pediatrist		2 (5.0)	

TD—typically developing, NDDs—neurodevelopmental disorders, SD—standard deviation, NA—not applicable.

**Table 2:** Participation in activities of daily living in children with and without NDDs (*n* = 40).

Contexts of Participation	TD Group	NDDs Group	Δ	*p*-Value
	Mean (SD)	Mean (SD)		
Home participation	97.7 (5.3)	76.7 (12.5)	4.0	<0.001
Community participation	96.3 (5.1)	68.1 (20.7)	5.5	<0.001
School participation	97.8 (5.3)	77.5 (12.4)	3.8	<0.001
Home and community instrumental living activities	88.8 (17.4)	59.2 (22.7)	1.7	<0.001
CASP total score	95.6 (5.6)	71.9 (13.4)	4.2	<0.001

TD—typically developing, NDDs—neurodevelopmental disorders, SD—standard deviation, Δ—effect size, CASP—Child and Adolescent Scale of Participation.

**Table 3:** Participation limitations in daily living in children with and without NDDs (*n* = 40).

**Mild Participation Limitations in at Least One ADL**	**TD Group**	**NDDs Group**	***p*-Value**	**OR (95% CI)**
	***N* (%)**	***N* (%)**		
Home participation	4 (20.0)	18 (90.0)	<0.001	36.0 (5.8–223.5)
Community participation	8 (40.0)	17 (85.0)	0.003	8.5 (1.9–38.8)
School participation	4 (20.0)	19 (95.0)	<0.001	76.0 (7.7–750.5)
Home and community instrumental living activities	10 (50.0)	20 (100.0)	<0.001	41.0 (2.2–770.1)
Overall (in at least one ADL)	12 (60.0)	20 (100.0)	0.008	27.9 (1.5–526.1)
**Moderate or Severe Participation Limitations in at Least One ADL**	**TD Group**	**NDDs Group**	***p*-Value**	**OR (95% CI)**
	***N* (%)**	***N* (%)**		
Home participation	1 (5.0)	13 (65.0)	<0.001	24.4 (3.6–154.2)
Community participation	1 (5.0)	13 (65.0)	<0.001	24.4 (3.6–154.2)
School participation	4 (20.0)	19 (95.0)	<0.001	47.7 (6.7–338.7)
Home and community instrumental living activities	4 (20.0)	12 (60.0)	0.010	5.4 (1.4–21.0)
Overall (in at least one ADL)	7 (35.0)	19 (95.0)	<0.001	23.4 (3.6–154.2)

ADL—activity of daily living, TD—typically developing, NDDs—neurodevelopmental disorders, OR—odds ratio, CI—confidence interval.

**Table 4:** Associations between the different contexts of participation (*n* = 40).

**Total Sample (*n* = 40)**			
**Contexts of Participation**	**Home Participation**	**Community Participation**	**School Participation**
Community participation	0.818 ***	-	-
School participation	0.856 ***	0.849 ***	-
Home and community instrumental living activities	0.731 ***	0.715 ***	0.642 ***
**TD Group (*n* = 20)**			
**Contexts of Participation**	**Home Participation**	**Community Participation**	**School Participation**
Community participation	0.416 ^†^	-	-
School participation	0.261	0.604 **	-
Home and community instrumental living activities	0.225	0.484 *	0.021
**NDDs Group (*n* = 20)**			
**Contexts of Participation**	**Home Participation**	**Community Participation**	**School Participation**
Community participation	0.542 *	-	-
School participation	0.807 ***	0.641 **	-
Home and community instrumental living activities	0.469 *	0.441 ^‡^	0.488 *

^†^*p* = 0.068, ^‡^ *p* = 0.052, * *p* < 0.05, ** *p* < 0.01, *** *p* < 0.001.

## References

[1] American Psychiatry Association (2013). Diagnostic and Statistical Manual of Mental Disorders.

[2] Nowell K.P., Bodner K.E., Mohrland M.D., Kanne S.M., Brenner L.A., Reid-Arndt S.A., Elliott T.R., Frank R.G., Caplan B. (2019). Neurodevelopmental disorders. Handbook of Rehabilitation Psychology.

[3] Blackburn C., Read J., Spencer N., Lemer C., Todd K., Cheung R., Murphy O. (2013). Children with neurodevelopmental disabilities. Annual Report of the Chief Medical Officer 2012. Our Children Deserve Better: Prevention Pays.

[4] Arora N.K., Nair M.K.C., Gulati S., Deshmukh V., Mohapatra A., Mishra D., Patel V., Pandey R.M., Das B.C., Divan G. (2018). Neurodevelopmental disorders in children aged 2–9 years: Population-based burden estimates across five regions in India. PLoS Med..

[5] Dietrich K.N., Eskenazi B., Schantz S., Yolton K., Rauh V.A., Johnson C.B., Alkon A., Canfield R.L., Pessah I.N., Berman R.F. (2005). Principles and practices of neurodevelopmental assessment in children: Lessons learned from the Centers for Children’s Environmental Health and Disease Prevention Research. Environ. Health Perspect..

[6] Cleaton M.A.M., Kirby A. (2018). Why Do We Find it so Hard to Calculate the Burden of Neurodevelopmental Disorders?. J. Child. Dev. Disord..

[7] Polanczyk G.V., Willcutt E.G., Salum G.A., Kieling C., Rohde L.A. (2014). ADHD prevalence estimates across three decades: An updated systematic review and meta-regression analysis. Int. J. Epidemiol..

[8] Catala-Lopez F., Peiro S., Ridao M., Sanfelix-Gimeno G., Genova-Maleras R., Catala M.A. (2012). Prevalence of attention deficit hyperactivity disorder among children and adolescents in Spain: A systematic review and meta-analysis of epidemiological studies. BMC Psychiatry.

[9] Mariño M.C., Ageitos A.G., Álvarez J.A., Del Río Garma M., Cendón C.G., Castaño A.G., Nieto J.P. (2018). Prevalencia de trastornos del neurodesarrollo, comportamiento y aprendizaje en Atención Primaria. An. Pediatría.

[10] Pérez-Crespo L., Canals-Sans J., Suades-González E., Guxens M. (2020). Temporal trends and geographical variability of the prevalence and incidence of attention deficit/hyperactivity disorder diagnoses among children in Catalonia, Spain. Sci. Rep..

[11] Blank R., Barnett A.L., Cairney J., Green D., Kirby A., Polatajko H., Rosenblum S., Smits-Engelsman B., Sugden D., Wilson P. (2019). International clinical practice recommendations on the definition, diagnosis, assessment, intervention, and psychosocial aspects of developmental coordination disorder. Dev. Med. Child. Neurol..

[12] Delgado-Lobete L., Montes-Montes R., Pértega-Díaz S., Santos-del-Riego S., Cruz-Valiño J.M., Schoemaker M.M. (2020). Interrelation of Individual, Country and Activity Constraints in Motor Activities of Daily Living among Typically Developing Children: A Cross-sectional Comparison of Spanish and Dutch Populations. Int. J. Environ. Res. Public Health.

[13] Montes-Montes R., Delgado-Lobete L., Pereira J., Santos-del-Riego S., Pousada T. (2020). Psychometric Validation and Reference Norms for the European Spanish Developmental Coordination Disorder Questionnaire: DCDQ-ES. Int. J. Environ. Res. Public Health.

[14] Amador-Ruiz S., Gutierrez D., Martínez-Vizcaíno V., Gulías-González R., Pardo-Guijarro M.J., Sánchez-López M. (2018). Motor Competence Levels and Prevalence of Developmental Coordination Disorder in Spanish Children: The MOVI-KIDS Study. J. Sch. Health.

[15] Delgado-Lobete L., Santos-del-Riego S., Pértega-Díaz S., Montes-Montes R. (2019). Prevalence of suspected developmental coordination disorder and associated factors in Spanish classrooms. Res. Dev. Disabil..

[16] Morales-Hidalgo P., Roigé-Castellví J., Hernández-Martínez C., Voltas N., Canals J. (2018). Prevalence and Characteristics of Autism Spectrum Disorder Among Spanish School-Age Children. J. Autism Dev. Disord..

[17] Williams J.G., Higgins J.P., Brayne C.E. (2006). Systematic review of prevalence studies of autism spectrum disorders. Arch. Dis. Child..

[18] Baxter A.J., Brugha T.S., Erskine H.E., Scheurer R.W., Vos T., Scott J.G. (2015). The epidemiology and global burden of autism spectrum disorders. Psychol. Med..

[19] Kim Y.S., Leventhal B.L., Koh Y.J., Fombonne E., Laska E., Lim E.C., Cheon K.-A., Kim S.-J., Lee H., Song D.H. (2011). Prevalence of autism spectrum disorders in a total population sample. Am. J. Psychiatry.

[20] Bumin G., Günal A. (2008). The effects of motor and cognitive impairments on daily living activities and quality of life in autistic children. Eur. J. Paediatr. Neurol..

[21] Jasmin E., Couture M., McKinley P., Reid G., Fombonne E., Gisel E. (2009). Sensori-motor and daily living skills of preschool children with autism spectrum disorders. J. Autism Dev. Disord..

[22] Elbasan B., Kayihan H., Duzgun I. (2012). Sensory integration and activities of daily living in children with developmental coordination disorder. Ital. J. Pediatr..

[23] Van der Linde B.W., van Netten J.J., Otten E., Postema K., Geuze R.H., Schoemaker M.M. (2015). Activities of Daily Living in Children with Developmental Coordination Disorder: Performance, Learning, and Participation. Phys. Ther..

[24] Volkan-Yazici M., Elbasan B., Yazici G. (2018). Motor Performance and Activities of Daily Living in Children with neurodevelopmental Disorders. Iran. J. Pediatr..

[25] World Health Organization (2001). International Classification of Functioning, Disability and Health: ICF..

[26] American Occupational Therapy Association (2014). Occupational therapy practice framework: Domain and process 3a ed. Am. J. Occup. Ther..

[27] Law M. (2002). Participation in the Occupations of Everyday Life. Am. J. Occup. Ther..

[28] Renée T. (2017). Kielhofner’s Model of Human Occupation: Theory and Application.

[29] Van der Linde B.W., van Netten J.J., Otten B.E., Postema K., Geuze R.H., Schoemaker M.M. (2014). Psychometric properties of the DCDDaily-Q: A new parental questionnaire on children’s performance in activities of daily living. Res. Dev. Disabil..

[30] Delgado-Lobete L., Montes-Montes R., van der Linde B.W., Schoemaker M.M. (2020). Assessment of Motor Activities of Daily Living: Spanish Cross-Cultural Adaptation, Reliability and Construct Validity of the DCDDaily-Q. Int. J. Environ. Res. Public Health.

[31] Bedell G. (2009). Further validation of the Child and Adolescent Scale of Participation (CASP). Dev. Neurorehabil..

[32] Rosenberg L., Bart O., Ratzon N.Z., Jarus T. (2013). Personal and Environmental Factors Predict Participation of Children With and Without Mild Developmental Disabilities. J. Child Fam. Stud..

[33] Magalhães L.C., Cardoso A.A., Missiuna C. (2011). Activities and participation in children with developmental coordination disorder: A systematic review. Res. Dev. Disabil..

[34] Liberman L., Ratzon N., Bart O. (2013). The profile of performance skills and emotional factors in the context of participation among young children with Developmental Coordination Disorder. Res. Dev. Disabil..

[35] De Schipper E., Lundequist A., Wilteus A.L., Coghill D., de Vries P.J., Granlund M., Holtmann M., Jonsson U., Karande S., Levy F. (2015). A comprehensive scoping review of ability and disability in ADHD using the International Classification of Functioning, Disability and Health-Children and Youth Version (ICF-CY). Eur. Child Adolesc. Psychiatry.

[36] De Schipper E., Lundequist A., Coghill D., de Vries P.J., Granlund M., Holtmann M., Jonsson U., Karande S., Levy F., Robison J.E. (2015). Ability and Disability in Autism Spectrum Disorder: A Systematic Literature Review Employing the International Classification of Functioning, Disability and Health-Children and Youth Version. Autism Res..

[37] Ferguson G.D., Jelsma J., Versfeld P., Smits-Engelsman B.C.M. (2014). Using the ICF Framework to Explore the Multiple Interacting Factors Associated with Developmental Coordination Disorder. Curr. Dev. Disord. Rep..

[38] Ibañez L.V., Kobak K., Swanson A., Wallace L., Warren Z., Stone W.L. (2018). Enhancing Interactions during Daily Routines: A Randomized Controlled Trial of a Web-Based Tutorial for Parents of Young Children with ASD. Autism Res..

[39] Schreibman L., Dawson G., Stahmer A.C., Landa R., Rogers S.J., McGee G.G., Halladay A. (2015). Naturalistic developmental behavioral interventions: Empirically validated treatments for autism spectrum disorder. J. Autism Dev. Disord..

[40] Smits-Engelsman B.C., Blank R., van der Kaay A.C., Mosterd-van der Meijs R., Vlugt-van den Brand E., Polatajko H., Wilson P.H. (2013). Efficacy of interventions to improve motor performance in children with developmental coordination disorder: A combined systematic review and meta-analysis. Dev. Med. Child Neurol..

[41] Offor N., Williamson P.O., Cacola P. (2016). Effectiveness of interventions for children with developmental coordination disorder in physical therapy contexts: A systematic literature review and meta-analysis. J. Mot. Learn. Dev..

[42] Preston N., Magallon S., Hill L.J., Andrews E., Ahern S.M., Mon-Williams M. (2016). A systematic review of high quality randomized controlled trials investigating motor skill programmes for children with developmental coordination disorder. Clin. Rehabil..

[43] Fernández-Andrés M.A., Pastor-Cerezuela G., Sanz-Cervera P., Tárraga-Mínguez R. (2015). A comparative study of sensory processing in children with and without Autism Spectrum Disorder in the home and classroom environments. Res. Dev. Disabil..

[44] Sanz-Cervera P., Pastor-Cerezuela G., González-Sala F., Tárraga-Mínguez R., Fernández-Andrés M.I. (2017). Sensory Processing in Children with Autism Spectrum Disorder and/or Attention Deficit Hyperactivity Disorder in the Home and Classroom Contexts. Front. Psychol..

[45] Barrios-Fernández S., Gozalo M., García-Gómez A., Romero-Ayuso D., Hernández-Mocholí M.Á. (2020). A New Assessment for Activities of Daily Living in Spanish Schoolchildren: A Preliminary Study of its Psychometric Properties. Int. J. Environ. Res. Public Health.

[46] Giménez-Nadal J.I., Molina J.A., Ortega R. (2017). Like my parents at home? Gender differences in children’s housework in Germany and Spain. Empir. Econ..

[47] Bedell G., Khetani M., Coster J., Law M., Cousins M., Majnemer A. (2012). Measures of participation in community, social and civic life for children with disabilities. Measures of Outcomes and Their Determinants for Children and Youth with Developmental Disabilities.

[48] Bedell G. The Child and Adolescent Scale of Participation (CASP)©. Administration and Scoring Guidelines. http://sites.tufts.edu/garybedell/files/2012/07/CASP-Administration-Scoring-Guidelines-8-19-11.pdf.

[49] Faul F., Erdfelder E., Lang A.-G., Buchner A. (2007). G*Power 3: A flexible statistical power analysis program for the social, behavioral, and biomedical sciences. Behav. Res. Methods.

[50] Gravetter F., Wallnau L. (2014). Essentials of Statistics for the Behavioral Sciences.

[51] Ferguson C.J. (2009). An effect size primmer: A guide for clinicians and researchers. Prof. Psychol. Res. Pract..

[52] Delgado-Lobete L., Pértega-Díaz S., Santos-del-Riego S., Montes-Montes R. (2020). Sensory Processing Patterns in Developmental Coordination Disorder, Attention Deficit Hyperactivity Disorder and Typical Development. Res. Dev. Disabil..

[53] Mazurek M.O., Petroski G.F. (2015). Sleep problems in children with autism spectrum disorder: Examining the contributions of sensory over-responsivity and anxiety. Sleep Med..

[54] Kuhaneck H.M., Britner P.A. (2013). A preliminary investigation of the relationship between sensory processing and social play in autism spectrum disorder. OTJR (Thorofare N. J.).

[55] Chien C.-W., Rodger S., Copley J., Branjerdporn G., Taggart C. (2016). Sensory Processing and Its Relationship with Children’s Daily Life Participation. Phys. Occup. Ther. Pediatr..

[56] Kirby A.V., Williams K.L., Watson L.R., Sideris J., Bulluck J., Baranek G.T. (2019). Sensory Features and Family Functioning in Families of Children With Autism and Developmental Disabilities: Longitudinal Associations. Am. J. Occup. Ther..

[57] Izadi-Najafabadi S., Ryan N., Ghafooripoor G., Gill K., Zwicker J.G. (2019). Participation of children with developmental coordination disorder. Res. Dev. Disabil..

[58] Harrowell I., Holien L., Lingam R., Emond A. (2018). The impact of developmental coordination disorder on educational achievement in secondary school. Res. Dev. Disabil..

[59] Poon K.K. (2011). The activities and participation of adolescents with autism spectrum disorders in Singapore: Findings from an ICF-based instrument. J. Intellect. Disabil. Res..

[60] Zendarski N., Mensah F., Hiscock H., Sciberras E. (2019). Trajectories of emotional and conduct problems and their association with early high school achievement and engagement for adolescents with ADHD. J. Atten. Disord..

[61] Kaljača S., Dučić B., Cvijetić M. (2019). Participation of children and youth with neurodevelopmental disorders in after-school activities. Disabil. Rehabil..

[62] Günal A., Bumin G., Huri M. (2019). The Effects of Motor and Cognitive Impairments on Daily Living Activities and Quality of Life in Children with Autism. J. Occup. Ther. Sch. Early Interv..

[63] Kreider C.M., Bendixen R.M., Young M.E., Prudencio S.M., McCarty C., Mann W.C. (2016). Social networks and participation with others for youth with learning, attention, and autism spectrum disorders. Can. J. Occup. Ther..

[64] Walton K.M. (2019). Leisure time and family functioning in families living with autism spectrum disorder. Autism.

[65] Şipoş R., Predescu E., Mureşan G., Iftene F. (2012). The Evaluation of Family Quality of Life of Children with Autism Spectrum Disorder and Attention Deficit Hyperactive Disorder. Appl. Med. Inform..

[66] Jellet R., Wood C.E., Giallo R., Seymour M. (2015). Family functioning and behaviour problems in children with Autism Spectrum Disorders: The mediating role of parent mental health. Clin. Psychol..

[67] Chien C.-W., Rodger S., Copley J. (2017). Parent-reported Participation in Children with Moderate-to-severe Developmental Disabilities: Preliminary Analysis of Associated Factors using the ICF Framework. Int. J. Disabil. Dev. Educ..

[68] Sikora D., Moran E., Orlinch F., Hall T.A., Kovacs E.A., Delahaye J., Clemons T.E., Kuhlthau K. (2013). The relationship between family functioning and behavior problems in children with autism spectrum disorders. Res. Autism Spectr. Disord..

[69] Soref B., Ratzon N.Z., Rosenberg L., Leitner Y., Jarus T., Bart O. (2012). Personal and environmental pathways to participation in young children with and without mild motor disabilities. Child Care Health Dev..

[70] Grajo L.C., Candler C., Sarafian A. (2020). Interventions within the Scope of Occupational Therapy to Improve Children’s Academic Participation: A Systematic Review. Am. J. Occup. Ther..

[71] Fox A., Dishman S., Valicek M., Ratcliff K., Hilton C. (2020). Effectiveness of Social Skills Interventions Incorporating Peer Interactions for Children with Attention Deficit Hyperactivity Disorder: A Systematic Review. Am. J. Occup. Ther..

[72] Cahill S.M., Egan B.E., Seber J. (2020). Activity- and Occupation-Based Interventions to Support Mental Health, Positive Behavior, and Social Participation for Children and Youth: A Systematic Review. Am. J. Occup. Ther..

[73] Cahill S.M., Beisbier S. (2020). Occupational Therapy Practice Guidelines for Children and Youth Ages 5-21 Years. Am. J. Occup. Ther..

[74] Crane L., Sumner E.M., Hill E.I. (2017). Emotional and behavioural prolems in children with Developmental Coordination Disorder: Exploring parent and teacher reports. Res. Dev. Disabil..

[75] Lingam R., Jongmans M.J., Ellis M., Hunt L.P., Golding J., Emond A. (2012). Mental Health Difficulties in Children with Developmental Coordination Disorder. Pediatrics.

[76] Glascoe F. (2000). Evidence-based approach to developmental and behavioural surveillance using parents’ concerns. Child Care Health Dev..

[77] Van der Linde B.W., Van Netten J.J., Otten E., Postema K., Geuze R.H., Schoemaker M.M. (2015). A systematic review of instruments for assessment of capacity in activities of daily living in children with developmental co-ordiantion disorder. Child Care Health Dev..

